# Self‐Assembled Soft Nanomaterials Via Silver(I)‐Coordination: Nanotube, Nanofiber, and Remarkably Enhanced Antibacterial Effect

**DOI:** 10.1002/advs.201500134

**Published:** 2015-07-14

**Authors:** Long Qin, Peng Wang, Yuanwang Guo, Chunying Chen, Minghua Liu

**Affiliations:** ^1^ Beijing National Laboratory for Molecular Science (BNLMS) CAS Key Laboratory of ColloidInterface and Chemical ThermodynamicsInstitute of ChemistryChinese Academy of SciencesBeijing100190P. R. China; ^2^ CAS Key Laboratory for Biomedical Effects of Nanomaterials and NanosafetyNational Center for Nanoscience and Technology of ChinaBeijing100190P. R. China

**Keywords:** antibacterial activity, antimicrobial agent, instant gelation, silver–metallogel, supramolecular self‐assembly

## Abstract

**Silver(I)‐induced instant gelation of pyridine‐containing Fmoc‐l‐glutamate** and its concentration‐dependent self‐assembly from nanotubes to nanofibers are investigated. The formed metallogel with nanostructure has remarkably enhanced antibacterial activities. Interestingly, the nanotube and nanofiber exhibit different antibacterial activities, and a corresponding antimicrobial mechanism is proposed.

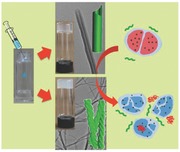

Self‐assembled soft nanomaterials via various noncovalent bonds have been attracting great interest due to their easy of fabrication and tailor‐made functions.^1^ Among various soft nanomaterials, metallogels^2^ that incorporate the metal ions into the supramolecular gel endowed the gel with certain unique features including the redox, mechano, magnetism, pH responsiveness,^3^ and new application potentials such as effective electron‐ and light‐emitting nanomaterials, asymmetric catalysis, visual chiral recognition, and chemosensors.^4^ While many of these functions were extensively investigated, the bioeffects of the gel materials were less reported.^5^ Among various metals, silver and its compounds (including silver nanomaterials) are historically well known and extensively investigated as antimicrobial agents to fight infections and control spoilage.^6^ Since the increasing of antibiotic‐resistant bacterial strains for the conventional antimicrobial treatments, free metal ions such as silver ions in large quantities are used, especially to fight against antibiotic‐resistant pathogens.^7^ Nowadays, a variety of silver‐containing biomedically relevant materials are exploited and used in clinical treatments including dental work, catheters, and burn wounds.^8^ It is expected to develop new materials with higher efficiency and less silver usage. The design and utility of silver‐coordinated metallogels, which might be able to show good stability, biocompatibility, and sustained effective antibacterial activity, come to our sight.^9^ Unfortunately, the antibacterial activity of these metallogels related to the microscopic nanostructures and the acting antibacterial mechanism have still not been completely understood. Herein, combining the structural features of metallogel and the good antimicrobial activity of silver(I)‐pyridyl coordination compounds, we report the silver(I) metallogels with tunable assembly structures from nanotubes to nanofibers and their excellent bioeffect as biocides to fight against either Gram‐positive or Gram‐negative bacteria. We also provide evidence that those metallogels have turned out to be the effective antimicrobial materials with the tunable inhibition activities. Meanwhile, the antimicrobial mechanism of metallogels incorporating different self‐assembled structures was also proposed.

The molecule *N*,*N*′‐bis(pyridyl‐4‐methyl)‐*N*‐fluorenyl‐9‐methoxycarbonyl(Fmoc)‐l‐glutamate (4MPFG, in **Scheme** **1**A) was synthesized via a condensation reaction between Fmoc‐Glu and 4‐(aminomethyl)pyridine, as shown in Figure S1 (Supporting Information). Although the compound is soluble in ethanol and cannot separately form the organogel, it showed instant gelation at room temperature as soon as equimolar amounts of AgNO_3_ aqueous solution were directly added under uniform stirring (*C*_4MPFG_ = 8 × 10^‐3^ m, titled as Gel 1 hereafter). Control experiment found that the mixing of same volume water with 4MPFG solution cannot induce gelation, suggesting the coordination between silver ions and pyridine group is crucial to the gel formation. Scanning electron microscopy (SEM) and transmission electron microscopy (TEM) characterization demonstrated that the well‐defined nanotubes with an outer diameter from ≈100 to 150 nm, and an inner diameter ≈20 nm were formed. It is very interesting to find that the self‐assembled behavior of the metallogel was largely dependent on the concentration of metallogels. As shown in **Figure** **1**, the self‐assembled nanostructures of 4MPFG/Ag^+^ metallogels obtained at different concentration underwent the great changes. At lower concentration (*C*_4MPFG_ = 4 × 10^‐3^ m), the nanotubes with the same unique property were formed, however varying the metallogel to higher concentration (from 8 to 16 × 10^‐3^ m) induced a transition of the self‐assembled nanostructures from nanotubes to the nanofibers. SEM and TEM investigation of metallogel at 16 × 10^‐3^ m (referred as Gel 2 hereafter) showed the entangled fibrillar structures with diameters ranging from 20 to 50 nm and micrometers in length were formed. More interestingly, this concentration‐dependent self‐assembly can be visually observed by a distinct variation on the gelation behavior from transparent gel at 4 × 10^‐3^ m to semitransparent gel at 8, 12 × 10^‐3^ m and finally to white gel at 16 × 10^‐3^ m, respectively. These formed instant metallogels consisting of 4MPFG and coordinated silver ions are very stable and can steadily stand upside down with holding a stirrer inside. The shape of the gels and mechanical strength can be maintained for at least six months. The rheological experiments of the metallogels at room temperature with different concentration were conducted in a typical frequency sweep mode with a constant strain 0.1% (Figure S2A, Supporting Information). It was found that the metallogels exhibited typical solid‐like rheological behavior with *G*′ an order of magnitude larger than *G*″ in the frequency range of 1–100 rad s^−1^. Interestingly, the storage modulus *G*′ and the loss modulus *G*″ greatly increased with the gel concentration, which suggested that the higher gel concentration, the much better mechanical rigidity. On the other hand, we further measured the sol–gel transition temperature (*T*_gel_) to characterize the thermal stability of these metallogels. *T*_gel_ also showed a concentration dependency, as shown in Figure S2B (Supporting Information). The *T*_gel_ obviously increased from 51 to 78 °C with the gel concentration from 4 to 16 × 10^‐3^ m.

**Scheme 1:**
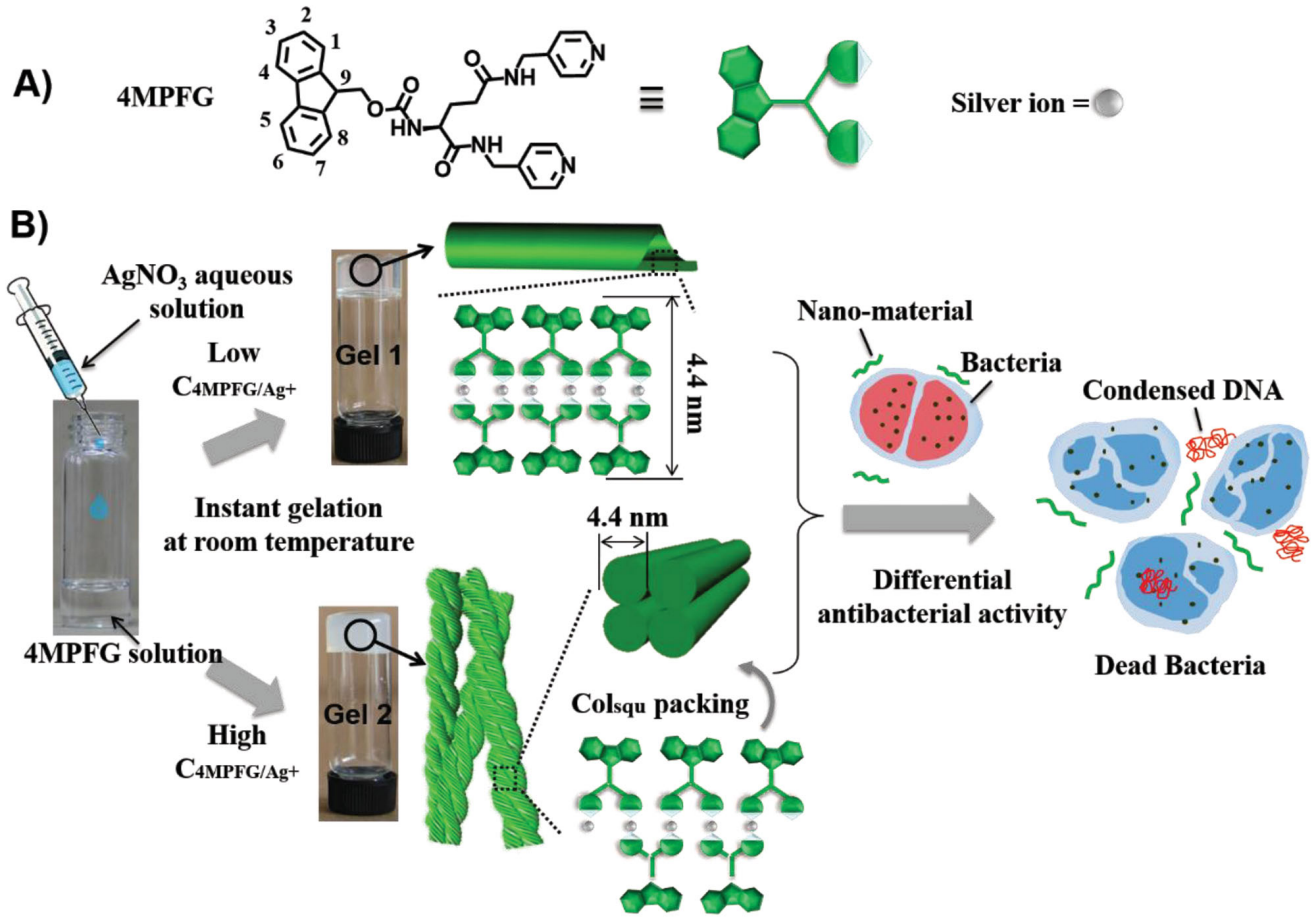
A) Molecular structure of 4MPFG. B) Schematic illustration of silver ion–induced instant gelation. The self‐assembly behavior of 4MPFG is highly dependent on the concentration of gelators and the self‐assembled nanotube and nanofiber structure can be obtained at different concentration. Thus, ring‐shaped and helical chain–type coordination mode is proposed. The formed nanomaterials show differential antibacterial activity when they are utilized as the antibiotic agents. The antimicrobial mechanism is that the nanomaterials can destroy the membrane integrity and induce DNA condensation to finally kill the bacteria.

**Figure 1:**
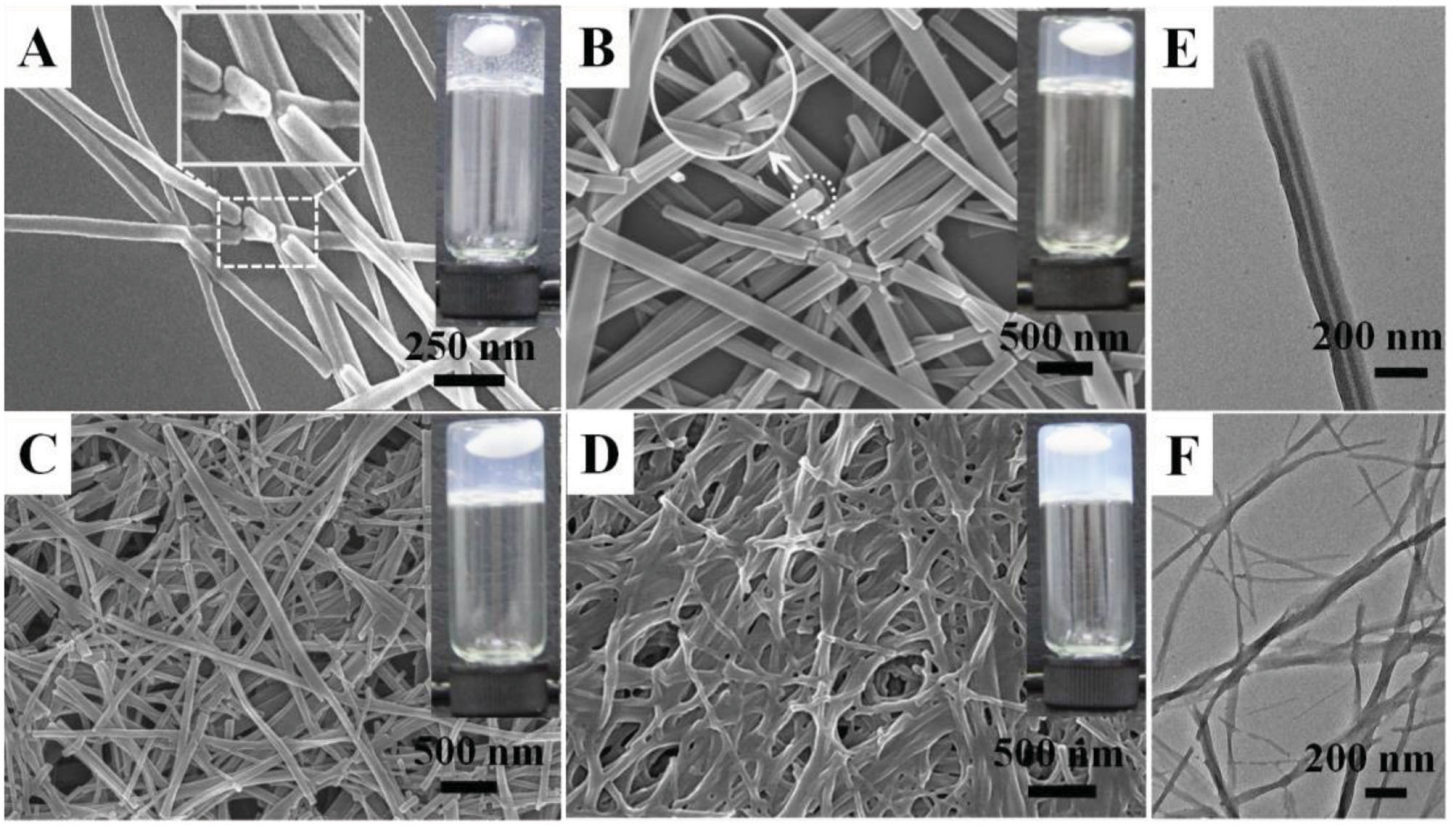
SEM and TEM images of the formed instant metallogels at different concentration: A–D) SEM of 4MPFG/Ag^+^ metallogels at the concentration of 4, 8, 12, and 16 × 10^‐3^ m and E,F) TEM of 4MPFG/Ag^+^ metallogels at the concentration of 8 and 16 × 10^‐3^ m, respectively.

The concentration‐dependent fluorescence spectra starting from very dilute solution to the assembling gel state have been studied to disclose the aggregation behavior of fluorene moieties within these metallogels (**Figure** **2**A). In the case of dilute solution, the emission peak of the fluorenyl moieties around 315 nm with the shoulder peak at 306 nm was the characteristic peak of the fluorenyl monomer. Fluorescence intensity slowly increases by increasing the concentration from 0.5 to 8 × 10^‐3^ m. This is due to the enhancement of concentration of gelator molecules. Meanwhile, gradual redshift of the fluorescence emission maximum from 315 to 323 nm was observed, during the gelator concentration increased to 8 × 10^‐3^ m, which suggest that the two fluorenyl moieties overlap in parallel fashion. Further enhancement of gelator concentration from 8 to 16 × 10^‐3^ m induced the emission peak continuously shifting to the long wavelength and finally located at 330 nm, which indicate the antiparallel packing of fluorenyl moieties.^10^ ^1^H NMR spectra of 4MPFG and 4MPFG with Ag^+^ at the concentration of 8 and 16 × 10^‐3^ m were measured in DMSO*‐d*_6_ solvent (Figure 2B). With the increase of concentration, the 2,7 and 3,6‐H signals of the fluorenyl moieties were gradually shifted downfield and 1,8 and 4,5‐H exhibited slightly upfield shift, which is the indication of antiparallel stacking of fluorenyl moiety.^11^ Moreover, the proton signals of pyridine ring showed downfield shift when Ag^+^ was added, which is due to the coordination between the pyridine and Ag^+^ imparting the electron‐withdrawing inductive effect on the proximate protons.^12^

**Figure 2:**
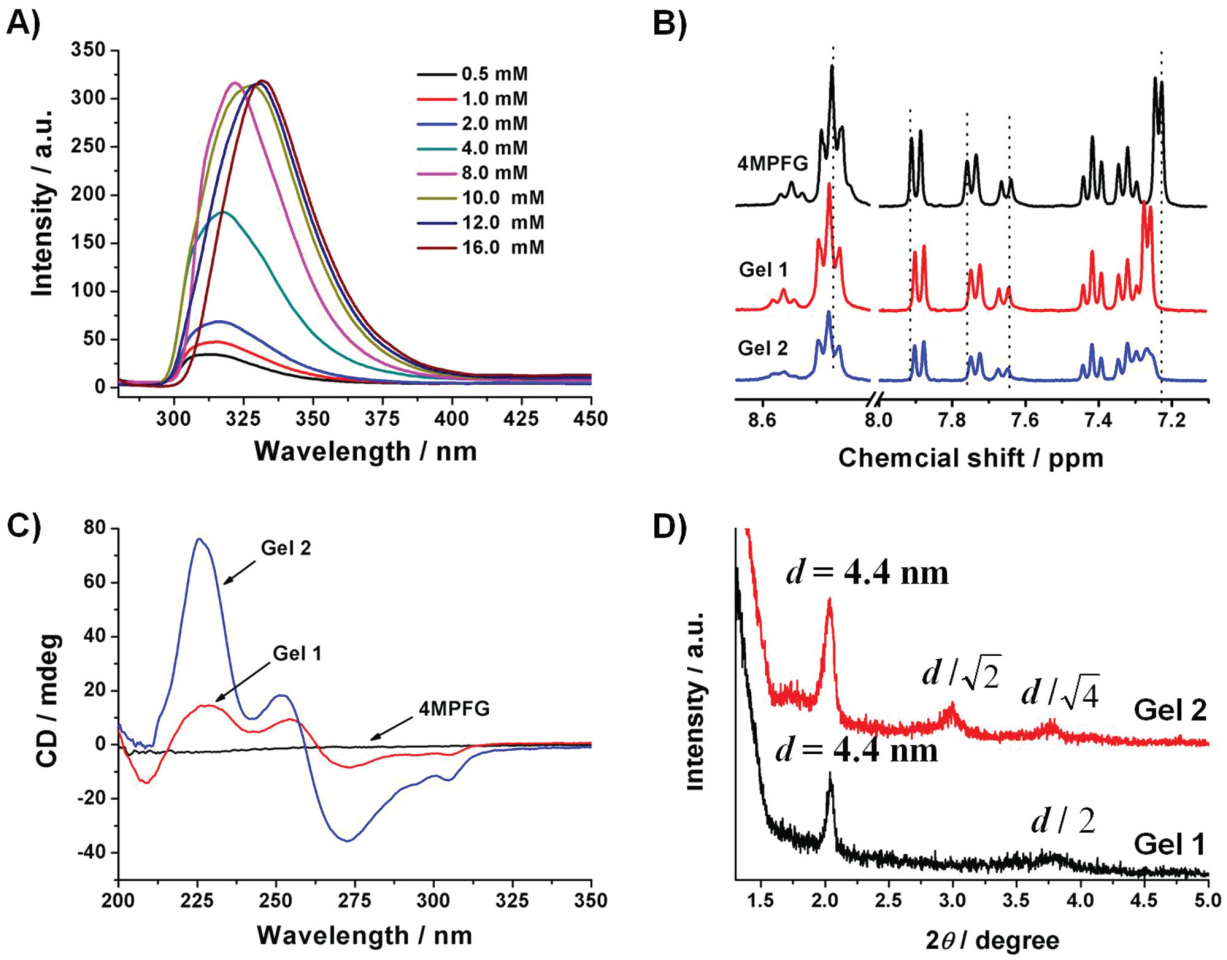
A) Concentration‐dependent fluorescence spectra of 4MPFG/Ag^+^ metallogels starting from very dilute solution (concentration 0.5 × 10^‐3^ m, *λ*_ex_ = 265 nm) to different gel state (concentration from 1 to 16 × 10^‐3^ m, *λ*_ex_ = 265 nm); B) ^1^H NMR spectra of 4MPFG, Gel 1 and Gel 2 in DMSO‐*d*_6_; C) CD; and D) XRD spectra of Gel 1 and Gel 2.

The supramolecular arrangement in Gel 1 and Gel 2 was further measured by circular dichroism (CD) spectroscopy since the gelator has a chiral center (Figure 2C). As expected, no obvious CD signal was detected in the solution of 4MPFG. However, the supramolecular chirality transfer from l‐glutamic acid to achiral fluorenyl groups and pyridine rings was detected in both metallogels. The positive Cotton effect at 228 nm and negative at 209 nm in Gel 1 suggest the helical arrangement of amide groups.^13^ Meanwhile, the positive bands from 255 nm and the negative Cotton effect with a double minima at around 305 and 273 nm were observed, which indicate the superhelical packing of the pyridine and fluorenyl moieties, respectively.^10^ Similar CD signals of amide and fluorenyl moieties were obtained in Gel 2, but the intensity was obviously strengthened, which suggest that those parts of groups were packed more closely in Gel 2. Moreover, the positive CD band of pyridine moieties was shifted to 251, which may be due to the different packing mode of pyridine rings.

In order to further investigate the structure of assemblies, small‐angle X‐ray diffraction (XRD) spectra of the two xerogels were measured, as shown in Figure 2D. The well‐developed diffraction patterns for the assemblies and *d*‐spacing values were estimated based on the Bragg's equation. The lamellar packed structure with a layer distance of 4.4 nm was obtained in Gel 1. This *d*‐spacing value is longer than the calculated molecular length (as simulated ≈1.8 nm) and roughly equals the length of two 4MPFG molecules with coordinated silver ions. In the case of Gel 2, the diffraction patterns were observed at *d*‐spacing value of 4.4, 3, and 2.3 nm estimated in the ratio of 1:$\sqrt{1/2}$:$\sqrt{1/4}$, which is in good agreement with columnar square phase packing.^14^

Fromm et al. have previously reported a phenomenon about the concomitant crystallization of two polymorphs. Two supramolecular isomers, a ring and a helix, are isolated from the same mother liquor as a result of concentration effects.^15^ Based on their work and the above results, two different self‐assembly mechanisms due to the concentration effect can be proposed as illustrated in Scheme 1. In Gel 1, the relative concentration of 4MPFG is low and silver ions tend to coordinate with the minor gelators. Therefore, an oval‐shaped ring that the two 4MPFG molecules are bridged by two silver ions is apt to be formed. Since the π–π stacking of fluorenyl moieties and the hydrogen bonding interactions as the main driving force in orthogonal direction, the oval‐shaped ring adopted face‐to‐face packing and then crimped to form nanotube structures. On the other hand, in Gel 2, the concentration of 4MPFG is higher with respect to the Gel 1 and the major gelators will be linked by silver ions to form the helical coordination polymer chains. These coordination polymer chains benefited the antiparallel stacking of the fluorenyl groups and finally closely packed according to columnar square phase to form the twisted nanofibers.

To determine whether the biological effects of the metallogels are closely correlated to their structures, we evaluated the antibacterial activities of the synthesized xerogels against Gram‐positive bacteria *S. aureus* and *S. epidermidis*, and Gram‐negative bacteria *Escherichia coli* (*E. coli*) using broth inhibition assay (details are shown in the Experimental Section). As shown in **Figure** **3**A and Figure S3 (Supporting Information), with the equimolar amount of Ag(Ι) ion, the Gel 1 and Gel 2 show better bacteriostatic activities than AgNO_3_ or the mixture of AgNO_3_ and ligand to *S. epidermidis*, *E. coli*, and *S. aureus*. However, no significant change of antibacterial activity is observed between AgNO_3_ and the mixture of AgNO_3_ and ligand, which suggesting that the formation of nanostructure played a key role for the inhibition of bacteria growth. More interestingly, different antibacterial results to *S. epidermidis* and *E. coli* are obtained between Gel 1 and Gel 2. This may be due to the different self‐assembled nanostructure between Gel 1 and Gel 2. Silver sulfadiazine (SD‐Ag) has received widespread acceptance as a topical agent to control bacterial infection, especially in helping heal burn wounds.^16^ Therefore, herein we compared the effect of SD‐Ag and metallogels on the growth curve of *S. epidermidis*, *E. coli*, and *S. aureus*. To our surprise, the metallogels exhibit better antibacterial activities than SD‐Ag against all bacteria (Figure S4, Supporting Information). Moreover, Gel 2 has better antibacterial activities than Gel 1 to *S. epidermidis* and *E. coli*, which is in agreement with the broth inhibition assay. Together, these results suggest that the nanostructure of metallogel plays the critical role in their antibacterial activity.

**Figure 3:**
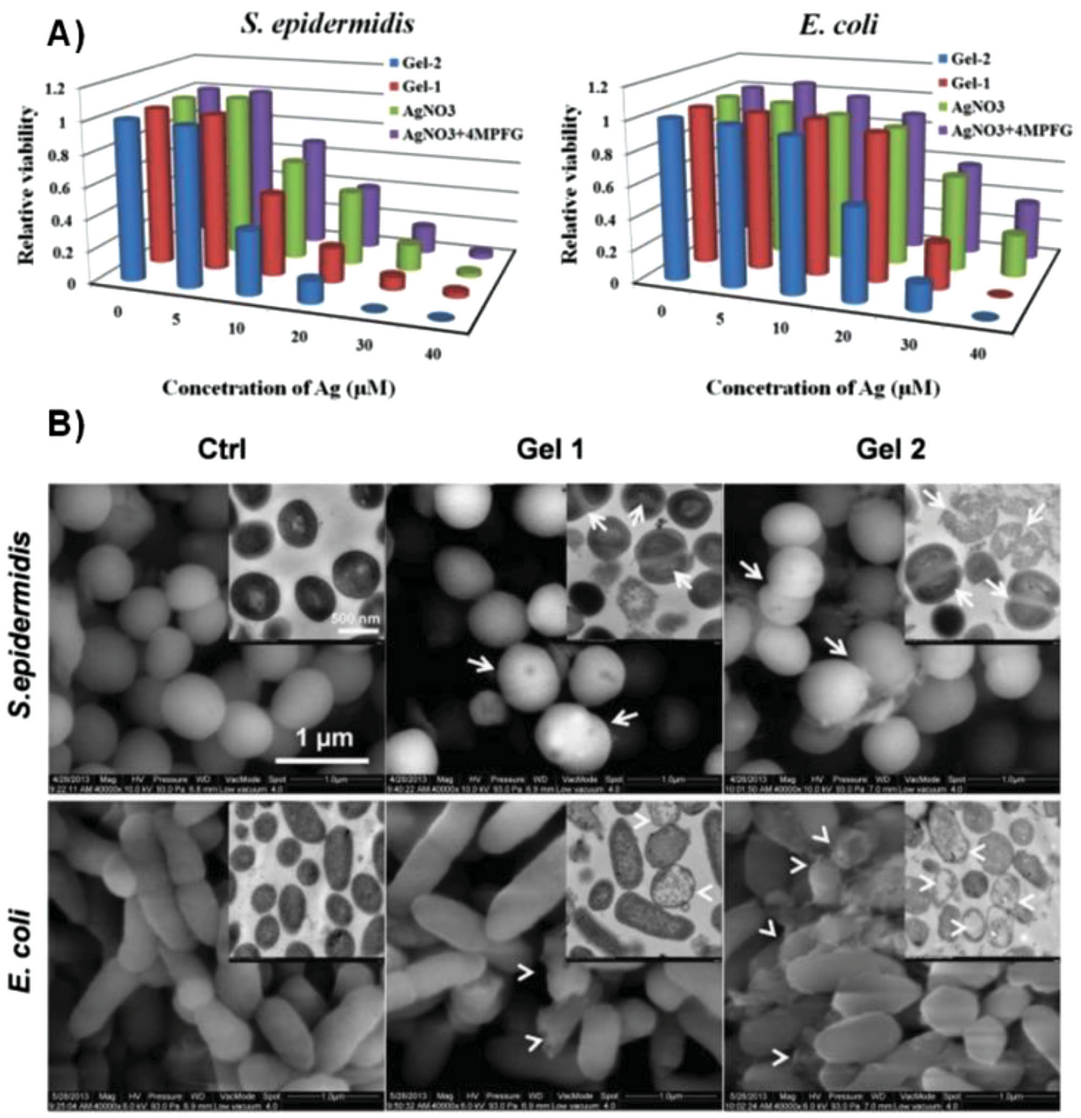
A) Microbicidal activities of Gel 1 (red), Gel 2 (blue), AgNO_3_ aqueous solution (green), AgNO_3_ and 4MPFG mixture aqueous solution (purple) against *S. epidermidis* and *E. coli*. B) Metallogel‐induced morphological change of *S. epidermidis* and *E. coli* via ESEM and TEM (insets) observations. *S. epidermidis* and *E. coli* were treated with Gel 1 or Gel 2 (concentration of Ag: 20 × 10^‐6^ m) for 12 h, while untreated groups were used as the control (Ctrl). The white arrows point to the cytokinesis‐blocked *S. epidermidis* cells. The white arrowheads point to the impaired *E. coli* cells.

To further explore the antibacterial mechanisms of metallogel, we used environmental scanning electronic microscopy (ESEM) and TEM to investigate the detailed ultrastructural changes caused by metallogels within the cell, such as the loss of membrane integrity, DNA condensation, and cytoplasmic reorganization. As shown in Figure 3B and Figure S5 (Supporting Information), significant morphological changes occurred in *S. epidermidis*, *E. coli*, and *S. aureus* cells after the addition of Gel 1 or Gel 2. The loss of membrane integrity can be clearly observed via ESEM and TEM in *S. epidermidis* and *E. coli* cells exposed to low‐dose Gel 1 and Gel 2. Moreover, there are intracellular substances released from some of the *S. epidermidis* and *E. coli* cells treated with high‐dose Gel 1 (Figure S6, Supporting Information). Importantly, the leakage of intracellular substances can be more obviously observed in cells treated with Gel 2, which provides one explanation for why Gel 2 has better antibacterial activities than Gel 1.

As examined by TEM, the untreated *S. epidermidis* and *E. coli* cells show unanimous electron density, and the DNA molecules are distributed randomly in almost all parts of the cells, suggesting that the cells are in a normal condition without environment disturbance.^17^ However, after treatment with Gel 1 or Gel 2, the cytoplasm membrane shrank or detached from the cell wall in *S. epidermidis* and *E. coli* cells. Moreover, there are many condensed DNA molecules positioned in the center of cells, which are indicated by white arrows. The replication of DNA molecules is effectively conducted only when DNA molecules are in a relaxed state. In a condensed form, DNA molecules lose their replicating abilities, thus the cytokinesis of cells will be blocked.^18^ In accord with these findings, there is a large increase of cytokinesis‐blocked *S. epidermidis* cells (indicated by white arrows) observed by both ESEM and TEM after the treatment of both Gel 1 and Gel 2. In addition, some *S. epidermidis* cells cannot finish their cell mitosis before they go to death. Therefore, metallogel‐caused DNA damage may be responsible for their antibacterial activities.

To further illustrate correlation between metallogel‐caused membrane disruption and the antibacterial activities of metallogels, we investigated the effect of metallogels on the permeability of cell membranes using the propidium iodide (PI) staining method. PI can intercalate within DNA and RNA to form a bright red fluorescent complex, but it cannot cross the membrane when the cell is alive. Therefore, intracellular staining of PI can specifically identify permeable cells. As shown in **Figure** **4** and Figure S7 (Supporting Information), both Gel 1 and Gel 2 induce serious permeability of cell membranes and leakage of nucleic acids in all bacteria. With an equimolar amount of Ag, the Gel 1 and Gel 2 induce more serious permeability of cell membranes and leakage of nucleic acids in all bacteria than the mixture of AgNO_3_ and ligand. Moreover, at a dose of 20 × 10^−6^ m Ag in Gel 1 and Gel 2, the percentage of permeable *S. epidermidis* with Gel 2 is nearly onefold higher than that with Gel 1; and the percentage of permeable *E. coli* with Gel 2 is also higher than that with Gel 1. These findings suggest that Gel 2 cause more damage to cell membrane of *S. epidermidis* and *E. coli* than Gel 1, which may be one reason why Gel 2 has better antibacterial activities than Gel 1 against *S. epidermidis* and *E. coli*.

**Figure 4:**
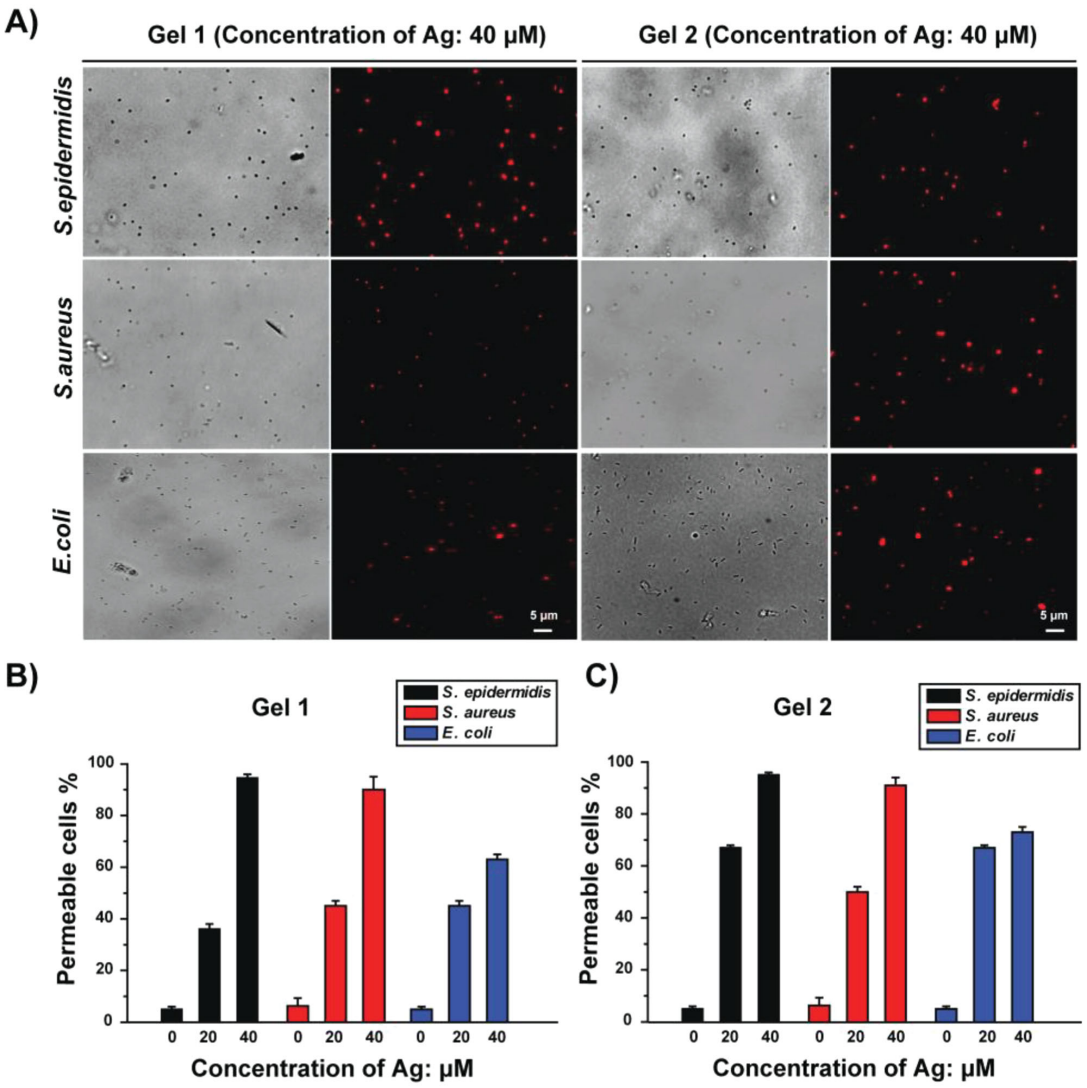
Monitoring metallogel‐induced permeability of cell membranes and leakage of nucleic acids via PI staining in *S. epidermidis*, *S. aureus*, and *E. coli*. A) Representative images of three types of bacteria treated with Gel 1 or Gel 2 (concentration of Ag: 40 × 10^‐6^ m) for 12 h. For each group of image, the left half shows an image in the differential interference contrast mode, while the right half shows the corresponding fluorescence image. B,C) *S. epidermidis*, *S. aureus*, and *E. coli* were treated with the indicated dose of Gel 1 or Gel 2, and then the percentage of cells with permeable membranes was calculated by counting three or four microscope fields from three independent experiments (each field includes 50–100 cells).

In summary, a bispyridyl‐conjugated Fmoc‐l‐glutamate is found to form instant gel at room temperature as soon as the incorporation of silver salt. The instant metallogel exhibits thermally reversible sol–gel transition and its self‐assembled behavior is largely dependent on the gelator concentration which result in distinct structural change from nanotube to nanofiber. The corresponding xerogels show good inhibitory activity against the growth of Gram‐positive and Gram‐negative bacteria and exhibit great potential to be utilized as better antibacterial reagents than SD‐Ag. Moreover, the metallogels with the two different self‐assembled nanostructures showed tunable antibacterial activities. Nanofibers may cause more damage to cell membrane than nanotubes and thus showed better antibacterial activities. The antibacterial results suggest that these 4MPFG/Ag^+^ metallogels may have valuable applications in various fields, such as the manufacture of household appliances and medical devices.

## Experimental Section

*Synthesis of 4MPFG*: All the starting reagents were purchased from commercial suppliers and used without further purified. *N*,*N*′‐bis(pyridin‐4ylmethyl)‐Fmoc‐l‐glutamate was synthesized by the amidation of Fmoc‐l‐glutamic acid with 4‐(aminomethyl)pyridine according to the following method. The compound Fmoc‐l‐glutamic acid (3.69 g, 0.01 mol) and 4‐(aminomethyl)pyridine (2.03 mL, 0.02 mol) with a catalytic amount of 1‐ethyl‐3‐(3‐dimethyllaminopropyl) carbodiimide hydrochloride (EDC·HCl, 5.75 g, 0.03 mol) and 1‐hydroxybenzotrizole (HOBt, 4.05 g, 0.03 mol) were mixed in dry CH_2_Cl_2_ (100 mL, 250 mL flask) and the reaction mixture was stirred for 72 h at room temperature. After the reaction, the solvent was removed by rotary evaporation. The resultant mixture was dissolved in 20 mL ethanol by heat and poured into 500 mL pure water. The precipitation was filtered and the crude product was obtained. After purification by silica column chromatography (CH_2_Cl_2_/CH_3_OH = 10/1, *R*_f_ = 0.5), the target product was obtained as a white solid (4.82 g, 88% yield). ^1^H NMR (400 MHz, DMSO‐*d*_6_) *δ* = 8.49 (t, 1H), 8.45–8.47 (m, 5H), 7.89 (d, 2H), 7.74 (d, 2H), 7.64 (d, 1H), 7.40–7.43 (m, 2H), 7.30–7.34 (m, 2H), 7.24 (d, 4H), 4.21–4.37 (m, 7H), 4.03–4.08 (m, 1H), 2.26–2.33 (m, 2H), 2–2.04 (m, 1H), 1.83–1.88 (m, 1H). Matrix‐assisted laser desorption/ionization time‐of‐fight mass spectrometry (MALDI‐TOF‐MS): *m*/*z*: calcd. for C_32_H_31_N_5_O_4_: 549.62; found: 550.2 [M + H]^+^, 572.2 [M + Na]^+^. Elemental analysis calcd. (%) for C_32_H_31_N_5_O_4_: C 69.93, H 5.69, N 12.74; found: C 69.99, H 5.97, N 12.51.

*Apparatus and Measurements*: ^1^H NMR spectra were recorded on a Bruker AV400 (400 MHz) spectrometer. Mass spectral data were obtained by using a BIFLEIII MALDI‐TOF MS instrument. Elemental analysis was performed on a Carlo–Erba‐1106 Thermo‐Quest. CD spectra were obtained using JASCO J‐810 CD spectrophotometers. Rheological studies were achieved on a Discovery DHR‐1 rheometer (TA Instruments). The rheology experiments were performed at 25 °C using parallel plate geometry in a Peltier plate (40 mm diameter aluminum plates). Fluorescence spectra were measured on an F‐4600 fluorescence spectrophotometer using a xenon lamp as the excitation source. X‐ray diffraction (XRD) was achieved on a Rigaku D/Max‐2500 X‐ray diffractometer (Japan) with CuKα radiation (*λ* = 1.5406 Å), which was operated at 45 kV, 100 mA. SEM measurements were performed on a Hitachi S‐4800 FE‐SEM microscope. TEM images were obtained on a JEM‐1011 electron microscope operating at accelerating voltages of 200 kV. The fluorescence of PI staining was excited by a 559 nm laser and observed with a laser scanning confocal microscope (Olympus, FV1000‐IX81). The morphology of bacterial was characterized by environmental scanning electronic microscopy (Quanta 200 FEG) or biological transmission electron microscope (HT7700) at 80 kV.

*Metallogels Fabrication and Characterizations*: A series of ethanol solution of 4MPFG and AgNO_3_ aqueous solution with the concentration from 1 to 32 × 10^−3^ m were prepared. 0.5 mL 4MPFG solution was first added in a capped test tube. Then 0.5 mL AgNO_3_ aqueous solution with the corresponding concentration was added into the above solution. The metallogels were instantly formed and incubated at 60 °C for 5 min under the darkness. The sealed test tube was then allowed to cool down to the room temperature. The formed metallogels were then washed by pure water for three times to remove the uncoordinated silver ions and separated by centrifuge at 10 000 rpm and finally dried under vacuum for 24 h to obtain the corresponding xerogels. For the TEM and SEM measurements of gel morphology, a small amount of dilute metallogels were placed onto a carbon‐coated copper grid (unstained) or a single‐crystal silicon plate (Pt coated), respectively, after being vacuum dried for 12 h. In the case of preparing samples for XRD measurements, gels were cast onto glass plates and dried under vacuum. In the process of measuring the CD and fluorescence spectra of metallogels, a quartz cuvette with 0.1 mm width was used.

*Determination of Antibacterial Activities of Metallogels*: In the broth inhibition assay, bacteria were cultured in the nutrient broth medium (5 g L^−1^ NaCl, 10 g L^−1^ tryptone powder, and 5 g L^−1^ beef extract powder, pH = 7.2) at 37 °C on a shaker bed at 200 rpm for 8–10 h and diluted with the broth to an optical density of 0.3 at 600 nm measured with UV–vis spectroscopy (Varian‐Cary Bio100), then we added 200 μL of each aqueous solution of Gel 1, Gel 2, AgNO_3_, AgNO_3_ + 4MPFG, or water into 4.8 mL of diluted broth containing bacteria in test tubes and cultured them at 37 °C on a shaker bed at 200 rpm for another 12 h. Finally, 2 mL of each mixture after incubation were transferred into a cuvette, and the OD was read with UV–vis spectroscopy at 600 nm against a reagent blank treated in the same manner. For the growth curve experiments, bacteria were cultured as above and diluted to an optical density of 0.15 at 600 nm, and then 10 μL of each aqueous solution of Gel 1, Gel 2, SD‐Ag, or water were added into 250 μL of diluted broth containing bacteria in Corning 96‐well plate. OD_600 nm_ was examined at different time courses using Tecan infinite 200 multimode microplate readers. Cultures were prepared in triplicate, and all experiments were repeated twice or more.

*Examination of Permeability of Cell Membranes by Fluorescence Assay*: Bacteria suspension (with a 0.3 optical density at 600 nm) mixed with Gel 1, Gel 2, AgNO_3_ + 4MPFG at a final concentration of 20 or 40 × 10^−6^ m was cultured at 37 °C for 12 h on a shaker bed at 200 rpm, the suspension was collected by centrifugation at 8000 rpm for 3 min, and washed with phosphate‐buffered saline (PBS, 0.01 m, pH = 7.4) twice. These bacteria were used as fluorescence assay, ESEM and TEM samples. The bacterial suspensions were incubated with an equal volume of the propidium iodide solution (3 × 10^−6^ m in PBS) in the dark for 30 min at room temperature, washed with PBS twice, and 20 μL of samples placed on a glass slide with a glass coverslip. The control assay was performed without any treatment. The fluorescence excited by a 559 nm laser with a laser scanning confocal microscope (Olympus, FV1000‐IX81) was observed.

*Preparation for ESEM and TEM Samples*: Bacteria were prepared as above. For ESEM assay, metallogel‐treated bacterial samples were fixed in 2.5% glutaraldehyde in 0.1 m cacodylate buffer (pH = 7.4) for 24 h, dehydrated with series‐grade ethanol, and critical‐point dried in CO_2_. Finally, cells were scanned with an ESEM. For TEM assay, metallogel‐treated bacterial samples were first fixed in 2.5% glutaraldehyde in 0.1 m cacodylate buffer (pH = 7.4) for 24 h, washed with PBS twice, further fixed with 1% OsO_4_ in PBS for 1 h, dehydrated in a graded series of ethanol solutions, treated with propylene oxide, and embedded in Durcupan. ≈80 nm thick sections were cut, placed on carbon film supported by copper grids, stained with uranyl acetate and lead citrate, and observed with a biological transmission electron microscope (HT7700) at 80 kV.

## Supporting information

As a service to our authors and readers, this journal provides supporting information supplied by the authors. Such materials are peer reviewed and may be re‐organized for online delivery, but are not copy‐edited or typeset. Technical support issues arising from supporting information (other than missing files) should be addressed to the authors.

Supplementary
