# Supplementary material for: Self‐Assembled Soft Nanomaterials Via Silver(I)‐Coordination: Nanotube, Nanofiber, and Remarkably Enhanced Antibacterial Effect
Source: Adv Sci (Weinh). 2015 Jul 14;2(11):1500134. doi: 10.1002/advs.201500134 (PMC5115345; doi:10.1002/advs.201500134)
Supplement: Supplementary file 1 — Supplementary [file ADVS-2-0c-s001.pdf]

Copyright WILEY-VCH Verlag GmbH & Co. KGaA, 69469 Weinheim, Germany, 2015.

ADVANCED SCIENCE
Open Access

Supporting Information

for *Adv. Sci.*, DOI: 10.1002/advs.201500134

Self-Assembled Soft Nanomaterials Via Silver(I)-Coordination: Nanotube, Nanofiber, and Remarkably Enhanced Antibacterial Effect

Long Qin, Peng Wang, Yuanwang Guo, Chunying Chen, and Minghua Liu**

((Supporting Information can be included here using this template))

Copyright WILEY-VCH Verlag GmbH & Co. KGaA, 69469 Weinheim, Germany, 2013.

Supporting Information

Self-assembled Soft Nanomaterials via Silver(I)-Coordination: Nanotube, Nanofiber and Remarkably Enhanced Antibacterial Effect

Long Qin, Peng Wang, Yuanwang Guo, Chunying Chen, and Minghua Liu**

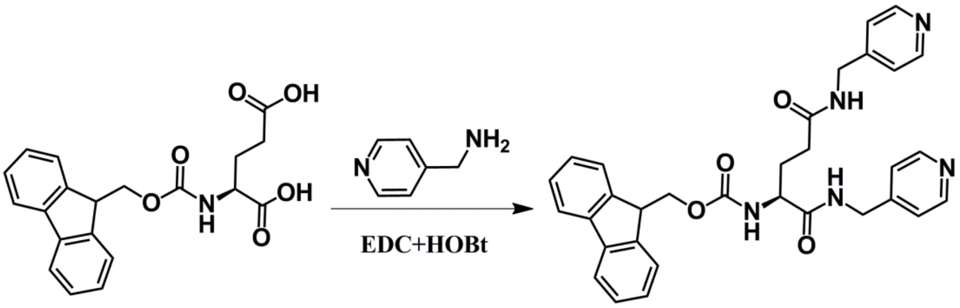

Figure S1. Synthetic route of 4MPFG.

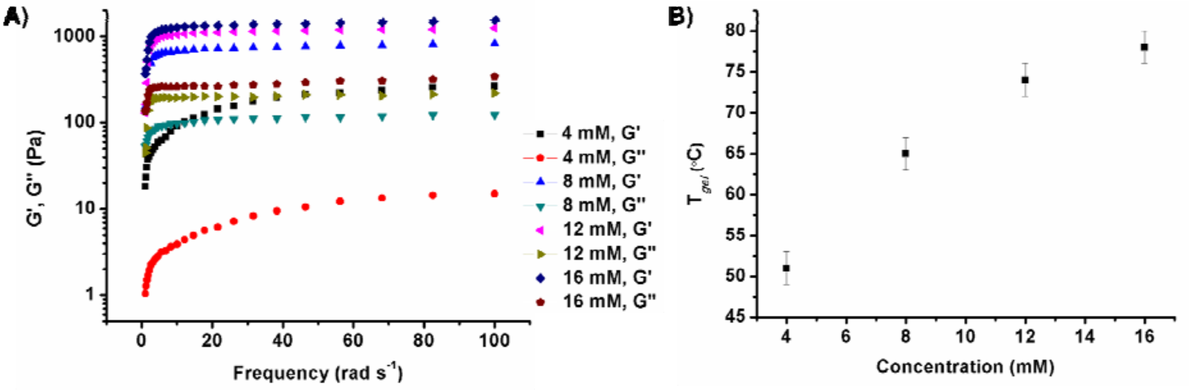

Figure S2. A) Frequency sweep rheometry of 4MPFG/Ag^{+} metallogels at a strain of 0.1% with different gel concentration from 4 to 16 mM. B) Plot of the T_{gel} as a function of gel concentration.

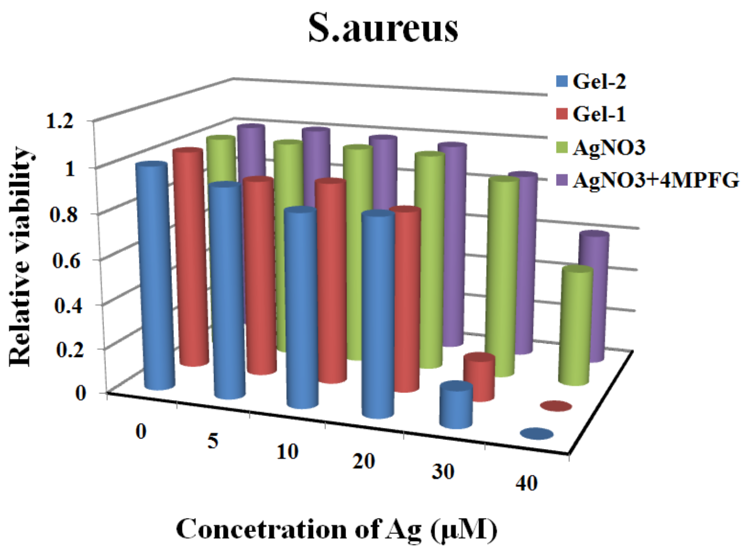

Figure S3. Microbicidal activities of Gel 1 (red), Gel 2 (blue), $AgNO_3$ aqueous solution (green), $AgNO_3$ and 4MPFG mixture aqueous solution (purple) against *S.aureus*.

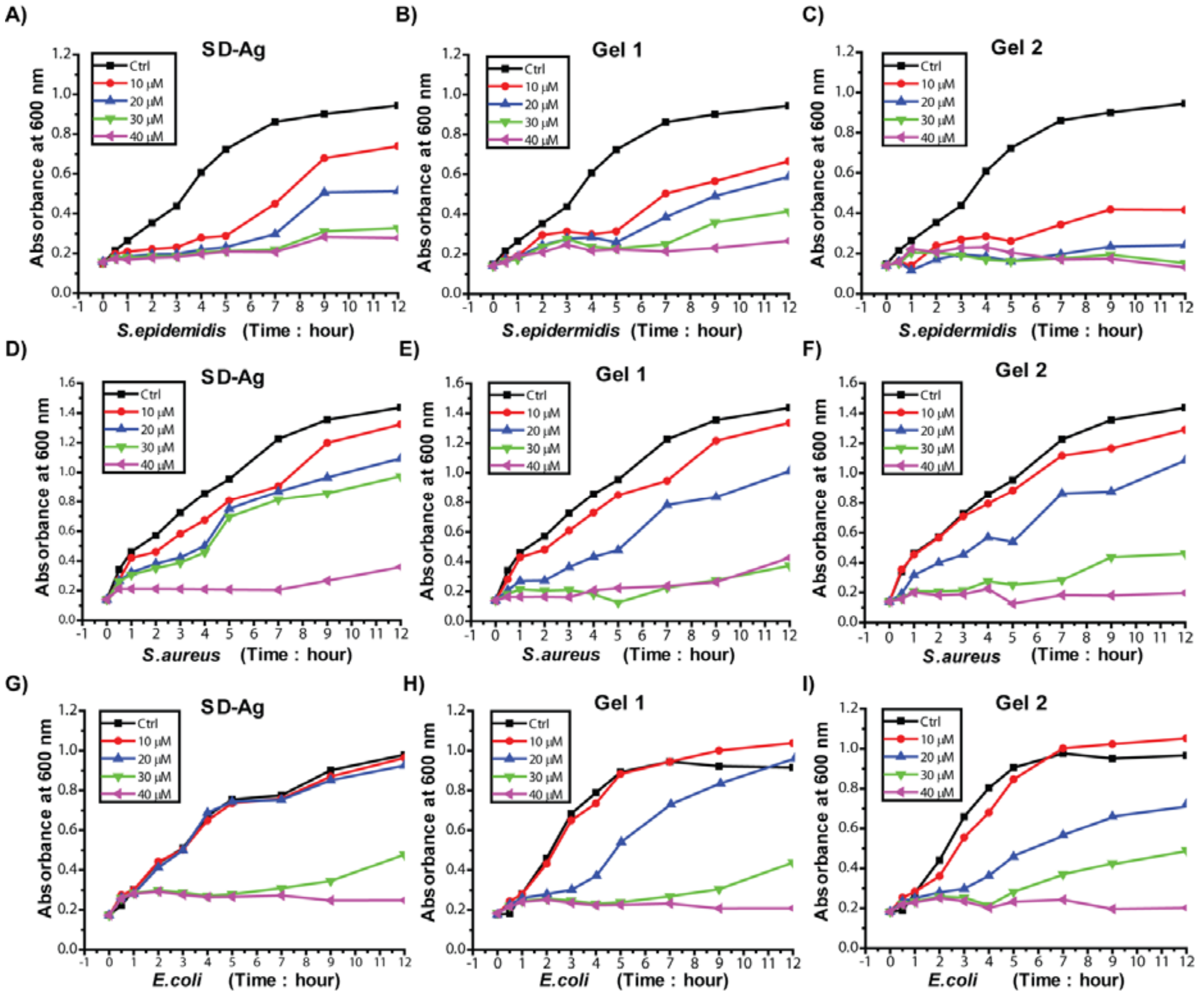

Figure S4. Effect of SD-Ag and metallogels on the growth curve of *S.epidermidis* (A-C), *S.aureus* (D-F) and *E.coli* (G-I).

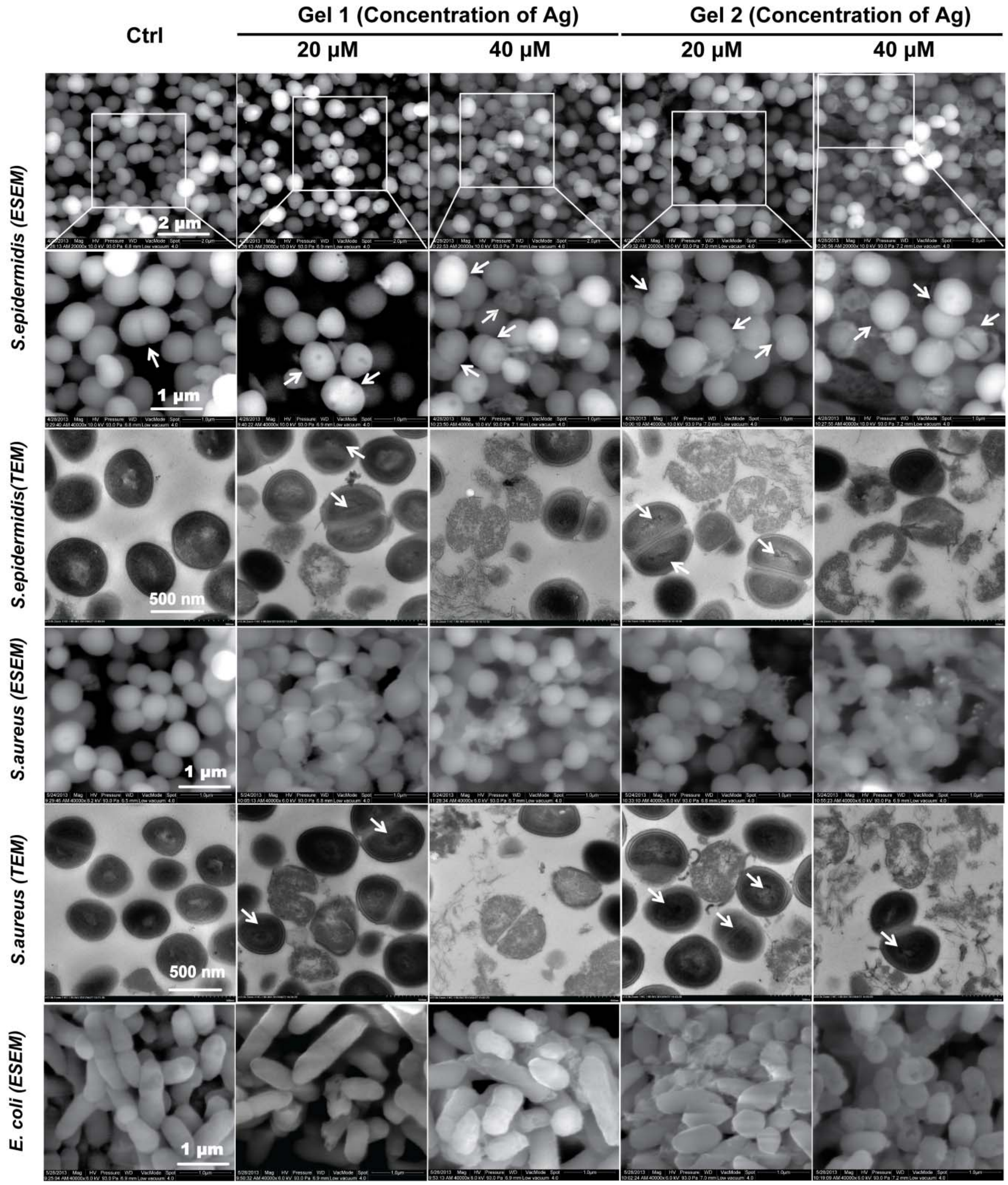

Figure S5. ESEM of *S. epidermidis*, *S. aureus* and *E. Coli* and TEM investigation of *S. epidermidis*, *S. aureus* under different concentration of Metallogels (Gel 1 and Gel 2) for 12 hours or control group (untreated). The white arrows point to the cytokinesis-blocked cells (ESEM) and the condensed DNA in cells (TEM).

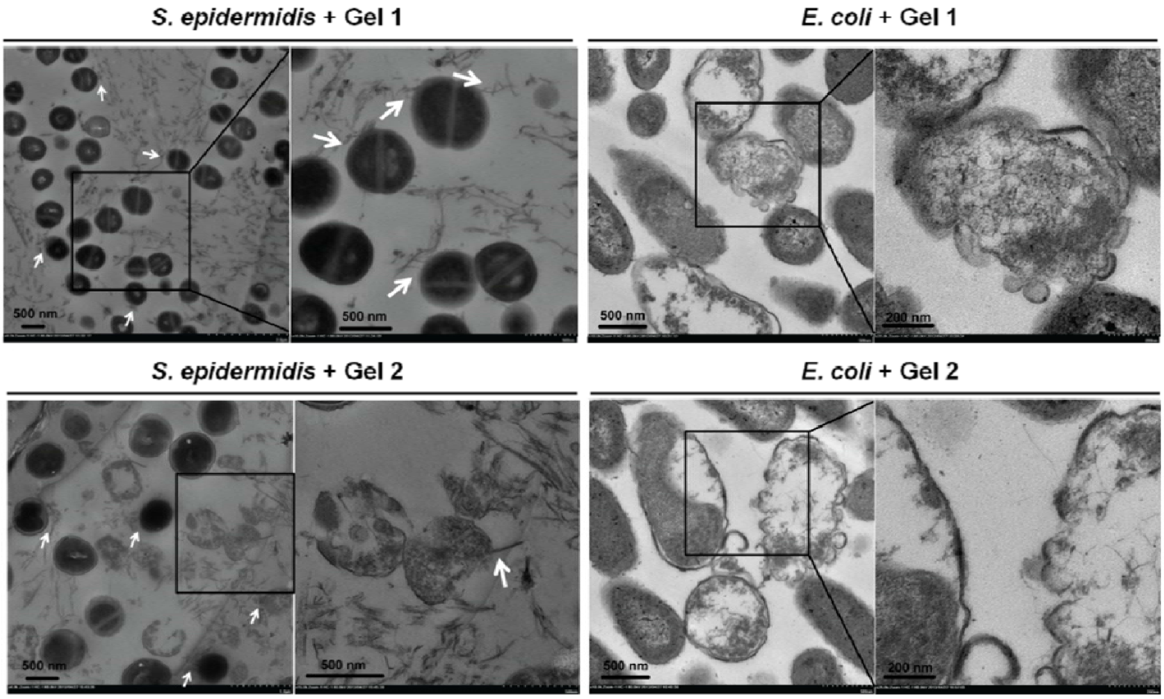

Figure S6. TEM observation of *S. epidermidis* and *E. Coli* treated with Gel 1 or Gel 2 (concentration of Ag at 40 μM) for 12 hours. The white arrows point to the intracellular substances released from some of the S. epidermidis treated with high-dose Gel 1 or Gel 2.

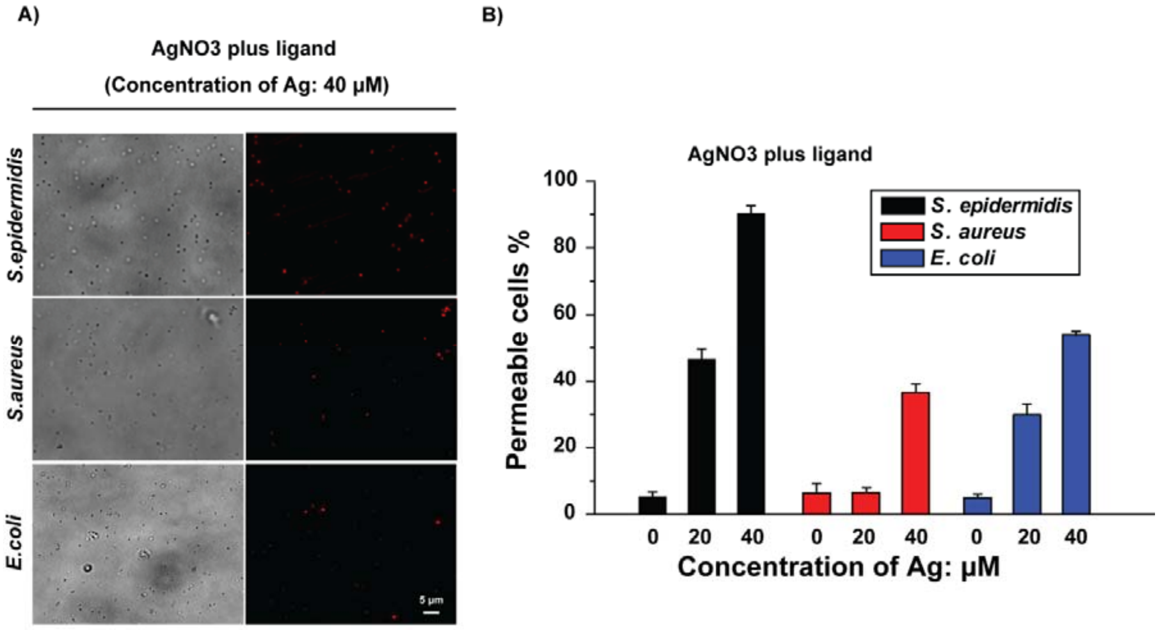

Figure S7. A) Representative images of three types of bacteria treated with $AgNO_3$+4MPFG (concentration of Ag: 40 μM) for 12 hours. For each group of image, the left half shows an image in the differential interference contrast mode, while the right half shows the corresponding fluorescence image. B) *S. epidermidis*, *S. aureus* and *E. coli* were treated with the indicated dose of Gel 1 or Gel 2, and then the percentage of cells with permeable membranes was counted from three or four fields of view from three independent experiments (each field includes 50-100 cells).
